# Supplementary material for: Comparative Effectiveness of Non-Pharmacological and Pharmacological Treatments for Non-Acute Lumbar Disc Herniation: A Multicenter, Pragmatic, Randomized Controlled, Parallel-Grouped Pilot Study
Source: J Clin Med. 2025 Feb 12;14(4):1204. doi: 10.3390/jcm14041204 (PMC11856646; doi:10.3390/jcm14041204)
Supplement: Supplementary file 1 [file jcm-14-01204-s001.zip › File S1.pdf]

Supplementary Material S1. Exclusion criteria

1. Diagnosis of certain serious medical conditions that can cause low back or lower extremity radiation pain (such as spinal metastases of tumors, acute fractures, and vertebral dislocations)
2. Progressive neurological deficits or severe neurological symptoms
3. Pain caused by a non-spinal soft tissue condition (such as tumor, fibromyalgia, rheumatoid arthritis, and gout)
4. Other chronic medical conditions (such as stroke and myocardial infarction, kidney disease, diabetic neuropathy, dementia, epilepsy)
5. Current use of any steroids, immunosuppressants, psychiatric medications, or other medications that may affect the results of the study
6. Inappropriate or unsafe for acupuncture or nerve block: Individuals with a history of bleeding disorders, anticoagulant therapy, or uncontrolled diabetes mellitus
7. Individuals who have taken medications that may affect pain, such as non-steroidal anti-inflammatory drugs (NSAIDs), or who have undergone acupuncture within the last 5 days
8. Pregnant or nursing women, or those planning a pregnancy
9. Patients within 3 months of cervical spine surgery
10. Participation in another clinical trial ending within 1 month of the trial, or planning to participate in another clinical trial during participation in the trial and follow-up within 6 months of the enrollment date
11. Difficulties in signing the informed consent form
12. Other cases where the investigator determines that participation in the clinical study is not appropriate

Supplementary Material S2. Primary and secondary outcomes

4.1 Primary outcome

4.1.1 Oswestry Disability Index

The primary outcome of the study is the change from baseline to Week 9 in the Oswestry Disability Index (ODI), which is the primary endpoint. The ODI is a 10-item questionnaire designed to evaluate the extent of functional disability associated with low back pain. Each item is scored on a 6-point Likert scale from 0 to 5. A higher score indicates a more severe impairment. An authorized Korean ODI survey will be conducted[1]. ODI was measured five times: at baseline, at Week 5, and at three follow-ups (Week 9, Week 14, and Week 27).

4.2 Secondary outcome

4.2.1 Low back pain numeric rating scale (NRS)

The NRS will be used to assess the intensity of the low back pain over the past week. The NRS is a pain scale that translates a patient's subjective pain intensity into objective numeric values. The participants will choose a number that best describes their current level of discomfort (0 for no pain to 10 for the most severe discomfort imaginable)[2-4]. NRS will be measured at baseline and on a weekly basis throughout the intervention period, including three follow-ups, for a total of 11 measurements.

4.2.2 Low back pain and radiating leg pain visual analog scale (VAS)

For the VAS assessment, the participants will be asked to mark the level of pain they feel as a point on a 100-mm line, indicating no pain at one end and the most severe pain imaginable at the other. The patient marks the level of low back pain experienced in the past week as a point on the line[3, 5]. VAS will be measured at baseline and on a weekly basis throughout the intervention period, including three follow-ups, for a total of 11 measurements.

4.2.3 Patient Global Impression of Change (PGIC)

PGIC is a method that asks patients to subjectively rate their improvement on a 7-point Likert scale. Patients respond to the improvement in functional limitations after treatment on a 7-point Likert scale (1, very much improved; 2, much improved; 3, minimally improved; 4, no change; 5, minimally worse; 6, much worse; or 7, very much worse)[6]. PGIC is measured three times during the follow-up visits at Week 9, Week 14, and Week 27.

4.2.4 Short-Form-12 Health Survey version 2

The Short-Form-12 Health Survey, version 2 (SF-12 v2), is a questionnaire designed for the assessment of health-related quality of life (HRQoL), consisting of 12 items across eight domains (physical functioning, role-physical, bodily pain, general health, vitality, social functioning, role-emotional, and mental health). A higher score indicates a better health-related quality of life. This study utilized the Korean version of the SF-12, validated for both reliability and validity [3]. SF-12 is measured five times: at baseline, at Week 5, and at three follow-ups (Week 9, Week 14, and Week 27).

4.2.5 EuroQol-5 Dimension

The 5-Level EuroQol-5 Dimension (EQ-5D-5L) is a method that assesses health status from multiple aspects and then indirectly calculates the quality weight of a specific health condition using pre-assigned preference scores for each functional level, which is widely used to measure quality of life. The EQ-5D-5L consists of five items: mobility, self-care, usual activities, pain, and anxiety/depression, each answered on a 5-point Likert scale. The total score is 0-1, with a higher score indicating a higher quality of life[7]. This study used the Korean version of the EQ5D-5L, which has been validated for reliability and validity[7]. EQ-5D-5L is measured five times: at baseline, at Week 5, and at three follow-ups (Week 9, Week 14, and Week 27).

4.2.6 *Credibility and expectancy*

A 9-point Likert scale was used to assess participant expectations of the treatment. At the screening visit, the participant candidates will answer the question, "How much do you think non-drug Korean Medicine treatments and Western medications will relieve your symptoms?" with a score (1 = not at all, 5 = somewhat, and 9 = very much).

References

1. Jeon, C.-H.; Kim, D.-J.; Kim, D.-J.; Lee, H.-M.; Park, H.-J. Cross-cultural adaptation of the Korean version of the Oswestry Disability Index (ODI). *J. Korean Soc. Spine Surg.* **2005**, *12*(2), 146–152. DOI: 10.4184/jkss.2005.12.2.146.

2. Karcioglu, O.; Topacoglu, H.; Dikme, O.; Dikme, O. A systematic review of the pain scales in adults: which to use? *Am. J. Emerg. Med.* **2018**, *36*(4), 707–714. DOI: 10.1016/j.ajem.2018.01.008.

3. Hawker, G.A.; Mian, S.; Kendzerska, T.; French, M. Measures of adult pain: Visual Analog Scale for Pain (VAS Pain), Numeric Rating Scale for Pain (NRS Pain), McGill Pain Questionnaire (MPQ), Short-Form McGill Pain Questionnaire (SF-MPQ), Chronic Pain Grade Scale (CPGS), Short Form-36 Bodily Pain Scale (SF-36 BPS), and Measure of Intermittent and Constant Osteoarthritis Pain (ICOAP) (sf-36 bps). *Arthritis Care Res.* **2011**, *63* (Suppl. 11), S240–S252. DOI: 10.1002/acr.20543.

4. Solodiuk, J.C.; Scott-Sutherland, J.; Meyers, M.; Myette, B.; Shusterman, C.; Karian, V.E.; Harris, S.K.; Curley, M.A.Q. Validation of the Individualized Numeric Rating Scale (INRS): a pain assessment tool for nonverbal children with intellectual disability. *Pain* **2010**, *150*(2), 231–236. DOI: 10.1016/j.pain.2010.03.016.

5. Daoust, R.; Beaulieu, P.; Manzini, C.; Chauny, J.-M.; Lavigne, G. Estimation of pain intensity in emergency medicine: a validation study. *Pain* **2008**, *138*(3), 565–570. DOI: 10.1016/j.pain.2008.02.007.

6. Dworkin, R.H.; Turk, D.C.; Farrar, J.T.; Haythornthwaite, J.A.; Jensen, M.P.; Katz, N.P.; Kerns, R.D.; Stucki, G.; Allen, R.R.; Bellamy, N.; et al. Core outcome measures for chronic pain clinical trials: IMMPACT recommendations. *Pain* **2005**, *113*(1–2), 9–19. DOI: 10.1016/j.pain.2004.09.012.

7. Kim, S.-H.; Ahn, J.; Ock, M.; Shin, S.; Park, J.; Luo, N.; Jo, M.-W. The EQ-5D-5L valuation study in Korea. *Qual. Life Res.* **2016**, *25*(7), 1845–1852. DOI: 10.1007/s11136-015-1205-2.
