# Supplementary material for: Comparative Effectiveness of Non-Pharmacological and Pharmacological Treatments for Non-Acute Lumbar Disc Herniation: A Multicenter, Pragmatic, Randomized Controlled, Parallel-Grouped Pilot Study
Source: J Clin Med. 2025 Feb 12;14(4):1204. doi: 10.3390/jcm14041204 (PMC11856646; doi:10.3390/jcm14041204)
Supplement: Supplementary file 1 [file jcm-14-01204-s001.zip › File S2.pdf]

Table S1. Study timeline.

Time point	Screening	Enrollment, allocation	Intervention				Follow-up			Unscheduled visit
	Week -1	Week 0	Week 1	Week 2, 3, 4	Week 5	Week 6, 7, 8	Week 9	Week 14	Week 27	
**Visit window**	**-10~0**	**Control point**	**±3**	**±3**	**±3**	**±3**	**±7**	**±14**	**±14**	
**Enrollment**										
Written informed consent	○									
Eligibility screening	○									
Vital signs	○	○	○	○	○	○	○	○	○	○
Sociodemographic characteristics	○									
Medical history	○	○								
Review of system	○									
L-spine MRI	○								○	
Blood analysis	○								○	
Randomized allocation		○								
**Interventions (Data collection only)**										
Treatment in non-pharmacological group (Experimental group)			○	○	○	○	○			
Treatment in pharmacological group (control group)			○	○	○	○	○			
**Assessments**										
Education for strategy		○								
Drug consumption	○	○	○	○	○	○	○	○	○	○
Adverse events		○	○	○	○	○	○	○	○	○
NRS of LBP			○	○	○	○	○	○	○	
NRS of leg pain	○		○	○	○	○	○	○	○	
VAS of LBP			○	○	○	○	○	○	○	
VAS of leg pain			○	○	○	○	○	○	○	
Oswestry Disability Index (ODI)			○		○		○	○	○	
PGIC							○	○	○	
SF-12			○		○		○	○	○	
EQ-5D-5L			○		○		○	○	○	
Physical sensorineural examination			○		○		○	○	○	
Credibility and expectancy	○									
Healthcare costs			○	○	○	○	○	○	○	

Non-healthcare costs		○ **(w2)**					
Loss of productivity	○	○	○	○	○	○	○
Compliance evaluation					○		

At Week -1, Week 0, and Week 1, this can be conducted on the same day.
If a blood test has been conducted within the last 2 weeks and a lumbar spine MRI has been performed after the onset of symptoms and within the last 3 months, the results can be used (including this as well as other hospitals). At the end of the study visit (Week 27), if the participants had undergone a blood test and lumbar spine MRI within the last 2 weeks from the visit date, the results could be used (including this hospital and other hospitals).
If the participants were unable to visit in Weeks 14 and 27, the questionnaires were completed over the phone or through the website.

Table S2. Cost calculation methods and data sources used in economic evaluation.

	Raw data	Type of costs	Estimation/calculation method
**Medical costs**			
Intervention (Week 2 to Week 9)	National Health Insurance (NHI) contribution, costs paid for using medical services in a medical institution	The total cost for each bill was calculated by adding all costs for each item (consultation fee for initial visit/subsequent visits, fee for examination/procedure/operation etc.)	For each participant, costs for each round of visit*frequency; Added by each round of visit
F/U			
KM medical institutions	Patient survey	Copayment and cost for non-NHI covered services	Amount indicated in the patient answer in the survey
	2022	Contribution paid by NHIS	HIRA Bigdata Open Portal (opendata.hira.or.kr)>Healthcare Statistics Information>Public Interest Disease Statistics: (Korean Medicine) lumbar disc herniation/intervertebral disc disorder
WM medical institutions	Patient survey	Copayment and cost for non-NHI covered services	Amount indicated in the patient answer in the survey
	2022	Contribution paid by NHIS	HIRA Bigdata Open Portal (opendata.hira.or.kr)>Healthcare Statistics Information>Public Interest Disease Statistics: lumbar disc herniation/intervertebral disc disorder
Medication costs	Unit cost based on 2022 values applied	Outpatient prescription	Unit cost*1-day dose *prescription days + Prescription and preparation fee (basic preparation fee, fee for prescription dispensing, medicine management fee, pharmacy management fee, medication guidance fee)
Other medical costs	Patient survey	Exercises, massages, etc.	Amount indicated in the patient answer in the survey
**Non-medical costs**			
Transportation cost	Patient survey	On foot/taxis/public transportation	Directly collected through the patient survey
Time cost	Patient survey		Collected information through the patient survey and direct measurement, and then the time was multiplied by wage
Productivity loss	Patient survey		WPAI questionnaire

*Calculations of transportation and time costs had errors during the survey process at one of the four hospitals (study institutions). Therefore, the average values of the other three variables were used as substitutes for hospitals with errors.

Table S3. Frequently used acupoints and types of Chuna Manual Therapy in the Non-pharmacological Treatment Group.

Category	Acupoints/Chuna	Total N=12	Frequency per patient
Acupoints	BL23 (Shenshu)	12 (100)	7.2±2
	BL54 (Zhibian)	9 (75)	7.1±2.3
	SP6 (Sanyinjiao)	8 (66.7)	7.1±2.5
	GB39 (Xuanzhong)	8 (66.7)	7.1±2.5
	GB30 (Huantiao)	4 (33.3)	7.8±0.5
	EX-B2 (Hyeopcheok)	3 (25)	8±0
	GV4 (Mingmen)	3 (25)	7.3±0.6
	BL26 Gyanyuanshu	2 (16.7)	8±0
	BL24 (Qihaishu)	2 (16.7)	8±0
	BL25 (Dachangshu)	2 (16.7)	8±0
	BL28 (Pangguangshu)	2 (16.7)	8±0
	BL27 (Xiaochangshu)	2 (16.7)	8±0
	BL36 (Chengfu)	2 (16.7)	8±0
	BL57 (Chengshan)	2 (16.7)	8±0
	ST36 (Zusanli)	2 (16.7)	8±0
Chuna	Prone position ilium adjustment method	4 (33.3)	8±0
	Lateral position lumbar vertebral distraction method	4 (33.3)	6.2±3.5
	Lumbar vertebral flexion distraction and adjustment method	3 (25)	8±0
	Lateral position lumbar vertebral adjustment method	3 (25)	8±0
	JS123	2 (16.7)	8±0
	Supine position cervical adjustment method	2 (16.7)	8±0
	Supine position thoracic vertebral adjustment method	2 (16.7)	8±0
	Lateral position lumbar vertebral distraction and adjustment	2(16.7)	8±0
	Prone position simple posterior inferior ilium adjustment	2(16.7)	7.5±0.7

Table S4. Frequently used medicines in the pharmacological treatment group.

Treatment	Description	n	Prescription days
Oral medications	Eperisone	16 (69.6)	20.8±15
	Aceclofenac	10 (43.5)	24.7±14
	Propionic acid derivatives	7 (30.4)	18.6±14.9
	Pregabalin	6 (26.1)	34±11.9
	Tramadol and paracetamol	5 (21.7)	18.4±12.3
	Esomeprazole	4 (17.4)	17.2±18.9
	Mosapride	4 (17.4)	24.5±18.2
	Other drugs for acid-related disorders	3 (13)	28.3±18.5
	Rebamipide	3 (13)	25±17.7
	Bromelains	2 (8.7)	9±7.1
	Dexibuprofen	2 (8.7)	16.5±13.4
	Famotidine	2 (8.7)	38±9.9
	Other anti-inflammatory and antirheumatic agents, non-steroids	2 (8.7)	11±14.1
Injectable medicines			
	Lidocaine	9 (39.1)	1.8±1.1
	Hyaluronidase	7 (30.4)	1.6±1.1
	Sodium chloride	5 (21.7)	2.6±2.5
	Dexamethasone	4 (17.4)	1.2±0.5
	Iohexol	3 (13)	1.7±1.2
	Iopamidol	3 (13)	1.3±0.6
	Carbohydrates	3 (13)	1.3±0.6
	Diclofenac	3 (13)	3.7±2.5
	Triamcinolone	2 (8.7)	2±1.4
	Other cicatrizants	2 (8.7)	2±1.4

Table S5. Primary and secondary outcome changes according to treatment group (ITT LMM).

	Baseline	Week 2	Week 3	Week 4	Week 5	Week 9	Week 14	Week 27
**NRS for LBP**								
**Non-PHM**	6.51 (6.10, 6.93)	5.54 (4.61, 6.48)	4.72 (3.76, 5.68)	4.54 (3.57, 5.50)	3.72 (2.76, 4.68)	2.45 (1.48, 3.41)	2.36 (1.39, 3.32)	2.36 (1.39, 3.32)
**PHM**		6.22 (5.53, 6.92)	5.56 (4.87, 6.24)	5.29 (4.61, 5.97)	5.24 (4.56, 5.92)	4.33 (3.65, 5.01)	4.65 (3.97, 5.33)	4.24 (3.56, 4.92)
**Difference***	**-**	0.68 (-0.52, 1.87)	0.84 (-0.38, 2.05)	0.75 (-0.46, 1.96)	1.52 (0.31, 2.73)	1.89 (0.68, 3.10)	2.30 (1.09, 3.50)	1.89 (0.68, 3.10)
**P value**	**-**	0.262	0.174	0.221	0.014*	0.003**	<0.001***	0.003**
**NRS for leg pain**								
**Non-PHM**	6.11 (5.74, 6.49)	5.12 (4.15, 6.09)	4.57 (3.57, 5.57)	4.03 (3.03, 5.02)	3.48 (2.48, 4.48)	2.03 (1.03, 3.02)	1.75 (0.76, 2.75)	2.21 (1.21, 3.20)
**PHM**		5.77 (5.05, 6.50)	5.16 (4.44, 5.88)	4.50 (3.78, 5.21)	4.50 (3.78, 5.21)	3.54 (2.83, 4.25)	4.00 (3.28, 4.71)	3.50 (2.78, 4.21)
**Difference***	**-**	0.65 (-0.59, 1.89)	0.59 (-0.66, 1.84)	0.47 (-0.78, 1.72)	1.02 (-0.23, 2.27)	1.52 (0.27, 2.77)	2.24 (0.99, 3.49)	1.29 (0.04, 2.54)
**P value**	**-**	0.297	0.35	0.455	0.109	0.018*	<0.001***	0.044*
**VAS for LBP**								
**Non-PHM**	61.46 (57.37, 65.54)	49.61 (39.34, 59.88)	43.52 (33.04, 54.01)	38.25 (27.76, 48.74)	32.80 (22.31, 43.29)	17.34 (6.85, 27.83)	16.26 (5.59, 26.94)	20.06 (9.39, 30.74)
**PHM**		58.25 (50.65, 65.85)	50.82 (43.29, 58.36)	47.68 (40.20, 55.15)	46.68 (39.20, 54.15)	35.54 (28.07, 43.01)	39.48 (31.88, 47.09)	33.16 (25.40, 40.92)
**Difference***	**-**	8.64 (-4.38, 21.66)	7.30 (-5.86, 20.46)	9.43 (-3.70, 22.55)	13.88 (0.76, 27.00)	18.20 (5.07, 31.32)	23.22 (9.88, 36.56)	13.10 (-0.33, 26.52)
**P value**	**-**	0.189	0.272	0.156	0.039*	0.007**	<0.001***	0.056
**VAS for leg pain**								
**Non-PHM**	66.77 (62.83, 70.72)	56.29 (46.65, 65.92)	51.31 (41.41, 61.21)	41.40 (31.50, 51.30)	37.58 (27.68, 47.48)	23.49 (13.59, 33.39)	22.34 (12.21, 32.47)	23.34 (13.21, 33.47)
**PHM**		63.13 (56.03, 70.24)	56.99 (49.97, 64.02)	53.95 (47.00, 60.89)	53.22 (46.27, 60.16)	44.49 (37.55, 51.44)	46.09 (38.97, 53.20)	41.75 (34.44, 49.07)
**Difference***	**-**	6.85 (-5.48, 19.17)	5.68 (-6.80, 18.16)	12.54 (0.11, 24.98)	15.64 (3.20, 28.07)	21.00 (8.56, 33.43)	23.75 (11.04, 36.45)	18.41 (5.59, 31.23)
**P value**	**-**	0.272	0.367	0.048*	0.014*	0.001**	<0.001***	0.005**
**ODI**								
**Non-PHM**	41.31 (35.88, 46.74)	-	-	-	28.88 (21.50, 36.27)	20.62 (13.24, 28.00)	21.00 (13.62, 28.39)	17.35 (9.97, 24.73)
**PHM**		-	-	-	31.67 (26.53, 36.81)	25.79 (20.65, 30.93)	25.44 (20.30, 30.58)	23.91 (18.77, 29.05)
**Difference***	-	-	-	-	2.79 (-6.39, 11.96)	5.17 (-4.00, 14.35)	4.43 (-4.74, 13.61)	6.57 (-2.61, 15.74)
**P value**	-	-	-	-	0.544	0.262	0.336	0.157
**EQ-5D-5L**								
**Non-PHM**	0.64 (0.58, 0.69)	-	-	-	0.75 (0.67, 0.82)	0.79 (0.72, 0.87)	0.82 (0.75, 0.89)	0.80 (0.73, 0.88)
**PHM**		-	-	-	0.72 (0.67, 0.77)	0.77 (0.72, 0.82)	0.76 (0.71, 0.81)	0.77 (0.72, 0.82)
**Difference***	-	-	-	-	-0.03 (-0.12, 0.06)	-0.02 (-0.11, 0.07)	-0.06 (-0.15, 0.03)	-0.04 (-0.13, 0.05)
**P value**	-	-	-	-	0.5	0.635	0.166	0.419
**PCS**								
**Non-PHM**	37.22 (35.20, 39.24)	-	-	-	79.35 (74.92, 83.77)	82.81 (78.39, 87.24)	82.40 (77.98, 86.82)	84.09 (79.67, 88.51)
**PHM**		-	-	-	78.28 (75.20, 81.36)	79.09 (76.01, 82.17)	77.77 (74.70, 80.85)	80.77 (77.69, 83.85)
**Difference***	-	-	-	-	-1.06 (-6.56, 4.43)	-3.73 (-9.22, 1.77)	-4.62 (-10.12, 0.87)	-3.32 (-8.81, 2.18)

**P value**	-	-	-	-	0.699	0.179	0.097	0.231
**MCS**								
**Non-PHM**	45.97 (42.67, 49.27)	-	-	-	94.59 (89.79, 99.38)	96.57 (91.78, 101.37)	97.15 (92.35, 101.94)	97.39 (92.59, 102.18)
**PHM**		-	-	-	92.52 (89.18, 95.87)	93.63 (90.29, 96.98)	94.37 (91.03, 97.72)	94.16 (90.82, 97.51)
**Difference***	**-**	-	-	-	-2.06 (-8.01, 3.89)	-2.94 (-8.89, 3.01)	-2.78 (-8.72, 3.17)	-3.22 (-9.17, 2.72)
**P value**	**-**	-	-	-	0.491	0.327	0.354	0.283
**PGIC**								
**Non-PHM**	**-**	-	-	-		1.93 (1.36, 2.50)	2.38 (1.82, 2.95)	2.48 (1.91, 3.04)
**PHM**	**-**	-	-	-		2.81 (2.41, 3.20)	3.03 (2.64, 3.43)	2.90 (2.50, 3.29)
**Difference***	**-**	-	-	-		0.88 (0.17, 1.58)	0.65 (-0.06, 1.36)	0.42 (-0.28, 1.13)
**P value**	**-**	-	-	-		0.016*	0.07	0.234

Table S6. Primary and secondary outcome changes according to the PP LMM treatment group.

		Baseline	Week 5	Week 9	Week 14	Week 27
NRS of LBP	**Non-PHM (n=11)**	6.52(6.08 to 6.95)	3.71 (2.73 to 4.70)	2.44 (1.46 to 3.42)	2.35 (1.37 to 3.33)	2.35 (1.37 to 3.33)
	**PHM (n=22)**		5.24 (4.56 to 5.93)	4.34 (3.65 to 5.02)	4.65 (3.97 to 5.34)	4.24 (3.56 to 4.93)
	**Difference***	-	1.53 (0.30 to 2.76)	1.89 (0.67 to 3.12)	2.30 (1.08 to 3.53)	1.89 (0.67 to 3.12)
	**P value**	-	0.015*	0.003**	<0.001***	0.003**
NRS of LEG	**Non-PHM (n=11)**	6.18(5.84 to 6.53)	3.54 (2.51 to 4.57)	2.08 (1.06 to 3.11)	1.81 (0.78 to 2.84)	2.27 (1.24 to 3.29)
	**PHM (n=22)**		4.56 (3.85 to 5.28)	3.61 (2.89 to 4.33)	4.06 (3.35 to 4.78)	3.56 (2.85 to 4.28)
	**Difference***	-	1.03 (-0.25 to 2.31)	1.53 (0.25 to 2.81)	2.25 (0.97 to 3.53)	1.30 (0.02 to 2.58)
	**P value**	-	0.114	0.020*	<0.001***	0.047*
VAS of LBP	**Non-PHM (n=11)**	63.09 (59.51 to 66.67)	34.07 (23.32 to 44.81)	18.61 (7.87 to 29.35)	17.53 (6.61 to 28.45)	21.33 (10.41 to 32.25)
	**PHM (n=22)**		48.24 (40.71 to 55.77)	37.10 (29.57 to 44.63)	41.04 (33.38 to 48.71)	34.72 (26.91 to 42.54)
	**Difference***	-	14.17 (0.82 to 27.53)	18.49 (5.13 to 31.85)	23.52 (9.95 to 37.09)	13.40 (-0.26 to 27.05)
	**P value**	-	0.038*	0.008**	<0.001***	0.054
VAS of LEG	**Non-PHM (n=11)**	67.27 (63.15 to 71.39)	37.98 (27.93 to 48.03)	23.89 (13.84 to 33.94)	22.74 (12.46 to 33.01)	23.74 (13.46 to 34.01)
	**PHM (n=22)**		53.68 (46.69 to 60.66)	44.95 (37.96 to 51.93)	46.54 (39.39 to 53.70)	42.21 (34.86 to 49.57)
	**Difference***	-	15.69 (3.12 to 28.27)	21.06 (8.48 to 33.63)	23.81 (10.96 to 36.65)	18.48 (5.52 to 31.43)
	**P value**	-	0.015*	0.001**	<0.001***	0.006**
ODI	**Non-PHM (n=11)**	41.99 (36.36 to 47.63)	29.57 (22.19 to 36.95)	21.31 (13.92 to 28.69)	21.69 (14.31 to 29.07)	18.03 (10.65 to 25.42)
	**PHM (n=22)**		32.36 (27.22 to 37.50)	26.48 (21.34 to 31.62)	26.12 (20.98 to 31.27)	24.60 (19.46 to 29.74)
	**Difference***	-	2.79 (-6.39 to 11.96)	5.17 (-4.00 to 14.35)	4.43 (-4.74 to 13.61)	6.57 (-2.61 to 15.74)
	**P value**	-	0.544	0.262	0.336	0.157
EQ5D5L	**Non-PHM (n=11)**	0.63(0.57 to 0.69)	0.74 (0.67 to 0.81)	0.79 (0.72 to 0.86)	0.82 (0.75 to 0.89)	0.80 (0.73 to 0.87)
	**PHM (n=22)**		0.71 (0.66 to 0.76)	0.77 (0.72 to 0.82)	0.75 (0.70 to 0.80)	0.76 (0.71 to 0.81)
	**Difference***	-	-0.03 (-0.12 to 0.06)	-0.02 (-0.11 to 0.07)	-0.06 (-0.15 to 0.03)	-0.04 (-0.13 to 0.05)
	**P value**	-	0.5	0.635	0.166	0.419
PCS	**Non-PHM (n=11)**		79.17 (74.75 to 83.59)	82.63 (78.21 to 87.06)	82.22 (77.80 to 86.64)	83.91 (79.49 to 88.33)

	**PHM (n=22)**	37.04 (34.92 to 39.16)	78.10 (75.02 to 81.18)	78.91 (75.83 to 81.99)	77.60 (74.52 to 80.68)	80.59 (77.52 to 83.67)
	**Difference***	-	-1.06 (-6.56 to 4.43)	-3.73 (-9.22 to 1.77)	-4.62 (-10.12 to 0.87)	-3.32 (-8.81 to 2.18)
	**P value**	-	0.699	0.179	0.097	0.231
**MCS**	**Non-PHM (n=11)**	45.69 (42.33 to 49.04)	94.30 (89.51 to 99.10)	96.29 (91.50 to 101.09)	96.87 (92.07 to 101.66)	97.11 (92.31 to 101.90)
	**PHM (n=22)**		92.24 (88.90 to 95.59)	93.35 (90.01 to 96.70)	94.09 (90.75 to 97.44)	93.88 (90.54 to 97.23)
	**Difference***	-	-2.06 (-8.01 to 3.89)	-2.94 (-8.89 to 3.01)	-2.78 (-8.72 to 3.17)	-3.22 (-9.17 to 2.72)
	**P value**	-	0.491	0.327	0.354	0.283
**PGIC**	**Non-PHM (n=11)**	-	-	1.93 (1.36 to 2.50)	2.38 (1.82 to 2.95)	2.48 (1.91 to 3.04)
	**PHM (n=22)**	-	-	2.81 (2.41 to 3.20)	3.03 (2.64 to 3.43)	2.90 (2.50 to 3.29)
	**Difference***	-	-	0.88 (0.17 to 1.58)	0.65 (-0.06 to 1.36)	0.42 (-0.28 to 1.13)
	**P value**	-	-	0.016*	0.07	0.234

Table S7. Primary and secondary outcome changes according to the treatment group.

		Baseline	Week 5	Week 9	Week 14	Week 27
**NRS of LBP**	**Non-PHM**	6.25 (5.34 to 7.16)	4.00 (2.82 to 5.18)	2.67 (1.64 to 3.70)	2.64 (1.54 to 3.73)	2.68 (1.40 to 3.96)
	**PHM**	6.65 (6.23 to 7.07)	5.09 (4.26 to 5.92)	4.21 (3.49 to 4.93)	4.50 (3.73 to 5.27)	4.07 (3.17 to 4.97)
	**Difference**	-	1.09 (-0.37 to 2.54)	1.54 (0.27 to 2.81)	1.86 (0.52 to 3.21)	1.38 (-0.19 to 2.96)
	**P value**	-	0.137	0.019*	0.008**	0.083
**NRS of LEG**	**Non-PHM**	6.08 (5.57 to 6.59)	3.82 (2.73 to 4.92)	2.36 (1.29 to 3.42)	2.05 (0.73 to 3.37)	2.57 (1.28 to 3.86)
	**PHM**	6.13 (5.62 to 6.64)	4.41 (3.63 to 5.18)	3.46 (2.71 to 4.21)	3.93 (3.00 to 4.86)	3.39 (2.49 to 4.30)
	**Difference**	-	0.58 (-0.77 to 1.93)	1.10 (-0.21 to 2.41)	1.88 (0.26 to 3.50)	0.82 (-0.76 to 2.41)
	**P value**	-	0.386	0.096	0.025*	0.299
**VAS of LBP**	**Non-PHM**	60.92 (54.35 to 67.49)	37.03 (25.90 to 48.17)	21.54 (10.24 to 32.83)	22.50 (8.34 to 36.66)	26.45 (11.11 to 41.79)
	**PHM**	61.74 (56.45 to 67.03)	46.44 (38.57 to 54.31)	35.32 (27.34 to 43.31)	38.30 (28.29 to 48.31)	30.80 (19.38 to 42.23)
	**Difference**	-	9.40 (-4.23 to 23.04)	13.79 (-0.05 to 27.63)	15.80 (-1.55 to 33.15)	4.35 (-14.81 to 23.51)
	**P value**	-	0.169	0.051	0.073	0.644
**VAS of LEG**	**Non-PHM**	62.92 (53.88 to 71.95)	40.98 (29.75 to 52.22)	26.70 (16.09 to 37.32)	27.35 (14.58 to 40.11)	28.61 (13.50 to 43.73)
	**PHM**	68.78 (65.14 to 72.42)	52.10 (44.22 to 59.97)	43.47 (36.03 to 50.91)	44.28 (35.31 to 53.24)	38.99 (27.81 to 50.18)
	**Difference**	-	11.11 (-2.76 to 24.98)	16.76 (3.66 to 29.87)	16.93 (1.19 to 32.67)	10.38 (-8.64 to 29.40)
	**P value**	-	0.112	0.014*	0.036*	0.272
**ODI**	**Non-PHM**	44.31 (38.12 to 50.51)	31.25 (24.78 to 37.71)	22.44 (15.34 to 29.55)	23.30 (15.03 to 31.58)	19.56 (12.09 to 27.04)
	**PHM**	39.74 (32.12 to 47.36)	31.40 (26.74 to 36.06)	25.91 (20.90 to 30.92)	25.32 (19.48 to 31.15)	23.83 (18.56 to 29.11)
	**Difference**	-	0.15 (-7.85 to 8.16)	3.46 (-5.26 to 12.19)	2.01 (-8.14 to 12.17)	4.27 (-4.91 to 13.45)
	**P value**	-	0.969	0.424	0.688	0.35
**EQ5D5L**	**Non-PHM**	0.62 (0.54 to 0.70)	0.72 (0.65 to 0.79)	0.77 (0.72 to 0.83)	0.80 (0.72 to 0.88)	0.78 (0.69 to 0.87)
	**PHM**	0.65 (0.57 to 0.72)	0.72 (0.67 to 0.77)	0.78 (0.74 to 0.82)	0.76 (0.71 to 0.82)	0.77 (0.71 to 0.83)
	**Difference**	-	0.00 (-0.08 to 0.08)	0.01 (-0.06 to 0.08)	-0.03 (-0.13 to 0.06)	-0.01 (-0.12 to 0.10)
	**P value**	-	0.98	0.829	0.488	0.844

**PCS**	**Non-PHM**	35.53 (32.68 to 38.37)	42.06 (38.08 to 46.04)	45.66 (41.56 to 49.75)	45.42 (40.86 to 49.98)	46.89 (42.33 to 51.45)
	**PHM**	38.10 (35.44 to 40.76)	41.88 (39.03 to 44.74)	42.79 (39.91 to 45.66)	41.39 (38.19 to 44.59)	44.49 (41.29 to 47.69)
	**Difference**	-	-0.18 (-5.12 to 4.77)	-2.87 (-7.92 to 2.18)	-4.04 (-9.66 to 1.59)	-2.40 (-8.02 to 3.22)
	**P value**	-	0.942	0.255	0.153	0.391
**MCS**	**Non-PHM**	46.14 (41.12 to 51.15)	46.84 (42.99 to 50.70)	49.17 (43.93 to 54.41)	49.50 (44.15 to 54.84)	50.09 (44.75 to 55.43)
	**PHM**	45.88 (41.51 to 50.25)	48.65 (45.87 to 51.43)	49.33 (45.63 to 53.03)	50.19 (46.41 to 53.97)	49.81 (46.04 to 53.58)
	**Difference**	-	1.81 (-2.95 to 6.56)	0.16 (-6.26 to 6.59)	0.70 (-5.86 to 7.25)	-0.28 (-6.83 to 6.27)
	**P value**	-	0.444	0.959	0.829	0.931
**PGIC**	**Non-PHM**	-	-	2.09 (1.60 to 2.58)	2.55 (1.99 to 3.11)	2.64 (1.96 to 3.32)
	**PHM**	-	-	2.73 (2.37 to 3.08)	2.95 (2.56 to 3.35)	2.82 (2.34 to 3.30)
	**Difference**	-	-	-0.64 (-1.24 to -0.03)	-0.41 (-1.09 to 0.28)	-0.18 (-1.01 to 0.65)
	**P value**	-	-	0.041*	0.233	0.659

Table S8. Primary and secondary outcome changes according to the treatment group.

		Baseline	Week 5	Week 9	Week 14	Week 27
**NRS of LBP**	**Non-PHM**	6.25 (5.34 to 7.16)	4.08 (2.80 to 5.36)	2.82 (1.70 to 3.95)	2.73 (1.53 to 3.93)	2.64 (1.27 to 4.01)
	**PHM**	6.65 (6.23 to 7.07)	5.10 (4.23 to 5.98)	4.17 (3.38 to 4.95)	4.50 (3.68 to 5.32)	4.08 (3.13 to 5.02)
	**Difference**	-	1.03 (-0.49 to 2.55)	1.34 (-0.05 to 2.74)	1.77 (0.34 to 3.20)	1.44 (-0.21 to 3.09)
	**P value**	-	0.178	0.059	0.017*	0.085
**NRS of LEG**	**Non-PHM**	6.08 (5.57 to 6.59)	3.85 (2.69 to 5.00)	2.33 (1.19 to 3.47)	2.19 (0.79 to 3.59)	2.55 (1.19 to 3.92)
	**PHM**	6.13 (5.62 to 6.64)	4.45 (3.64 to 5.26)	3.51 (2.71 to 4.31)	3.86 (2.90 to 4.82)	3.48 (2.51 to 4.45)
	**Difference**	-	0.61 (-0.82 to 2.03)	1.18 (-0.17 to 2.54)	1.67 (-0.04 to 3.37)	0.93 (-0.77 to 2.62)
	**P value**	-	0.392	0.085	0.055	0.273
**VAS of LBP**	**Non-PHM**	60.92 (54.35 to 67.49)	38.02 (25.92 to 50.11)	22.60 (10.45 to 34.74)	23.93 (9.10 to 38.76)	26.38 (10.40 to 42.37)
	**PHM**	61.74 (56.45 to 67.03)	46.39 (37.96 to 54.83)	35.60 (27.10 to 44.09)	39.64 (29.36 to 49.93)	32.95 (20.75 to 45.16)
	**Difference**	-	8.38 (-6.50 to 23.26)	13.00 (-1.76 to 27.76)	15.71 (-2.13 to 33.55)	6.57 (-13.08 to 26.22)
	**P value**	-	0.26	0.082	0.082	0.501
**VAS of LEG**	**Non-PHM**	62.92 (53.88 to 71.95)	41.04 (29.27 to 52.81)	27.55 (16.15 to 38.95)	29.26 (14.75 to 43.77)	30.20 (13.69 to 46.70)
	**PHM**	68.78 (65.14 to 72.42)	52.06 (43.70 to 60.41)	43.29 (35.29 to 51.30)	43.80 (33.74 to 53.86)	38.68 (26.19 to 51.16)
	**Difference**	-	11.02 (-3.22 to 25.25)	15.75 (1.97 to 29.52)	14.54 (-3.02 to 32.10)	8.48 (-12.95 to 29.91)
	**P value**	-	0.125	0.026*	0.101	0.426
**ODI**	**Non-PHM**	44.31 (38.12 to 50.51)	31.25 (24.78 to 37.71)	22.61 (14.54 to 30.69)	24.27 (15.05 to 33.49)	20.00 (11.66 to 28.33)
	**PHM**	39.74 (32.12 to 47.36)	31.40 (26.74 to 36.06)	26.10 (20.65 to 31.56)	25.53 (19.28 to 31.77)	23.51 (17.99 to 29.03)
	**Difference**	-	0.15 (-7.85 to 8.16)	3.49 (-6.12 to 13.10)	1.26 (-9.97 to 12.48)	3.51 (-6.62 to 13.64)
	**P value**	-	0.969	0.465	0.821	0.485
**EQ5D5L**	**Non-PHM**	0.62 (0.54 to 0.70)	0.72 (0.65 to 0.79)	0.77 (0.71 to 0.83)	0.79 (0.70 to 0.88)	0.79 (0.69 to 0.88)
	**PHM**	0.65 (0.57 to 0.72)	0.72 (0.67 to 0.77)	0.78 (0.73 to 0.82)	0.77 (0.71 to 0.83)	0.77 (0.70 to 0.84)
	**Difference**	-	0.00 (-0.08 to 0.08)	0.01 (-0.06 to 0.08)	-0.03 (-0.13 to 0.08)	-0.01 (-0.13 to 0.10)
	**P value**	-	0.98	0.799	0.611	0.805
**PCS**	**Non-PHM**	35.53 (32.68 to 38.37)	42.06 (38.08 to 46.04)	45.48 (40.93 to 50.03)	45.03 (39.93 to 50.12)	46.80 (41.77 to 51.84)

	**PHM**	38.10 (35.44 to 40.76)	41.88 (39.03 to 44.74)	42.91 (39.89 to 45.94)	41.34 (37.93 to 44.74)	44.56 (41.18 to 47.94)
	**Difference**	-	-0.18 (-5.12 to 4.77)	-2.57 (-8.03 to 2.89)	-3.69 (-9.96 to 2.58)	-2.24 (-8.40 to 3.91)
	**P value**	-	0.942	0.345	0.24	0.463
**MCS**	**Non-PHM**	46.14 (41.12 to 51.15)	46.84 (42.99 to 50.70)	49.33 (43.78 to 54.87)	49.91 (43.72 to 56.11)	50.06 (44.32 to 55.81)
	**PHM**	45.88 (41.51 to 50.25)	48.65 (45.87 to 51.43)	49.23 (45.32 to 53.15)	49.59 (45.32 to 53.87)	49.51 (45.55 to 53.48)
	**Difference**	-	1.81 (-2.95 to 6.56)	-0.09 (-6.83 to 6.65)	-0.32 (-7.74 to 7.10)	-0.55 (-7.64 to 6.55)
	**P value**	-	0.444	0.978	0.931	0.876
**PGIC**	**Non-PHM**	-	-	2.09 (1.60 to 2.58)	2.61 (2.00 to 3.22)	2.69 (1.95 to 3.43)
	**PHM**	-	-	2.73 (2.37 to 3.08)	2.94 (2.52 to 3.37)	2.84 (2.32 to 3.35)
	**Difference**	-	-	0.64 (0.03 to 1.24)	0.33 (-0.42 to 1.07)	0.15 (-0.75 to 1.04)
	**P value**	-	-	0.041*	0.376	0.739

Table S9. Area under the curve of each outcome by treatment group.

	Non-PHM	PHM	Difference (95% CI)	P value
**NRS of LBP**	83.65 (60.49 to 106.80)	118.00 (101.67 to 134.32)	-34.35 (-62.45 to -6.24)	0.018*
**NRS of LEG**	74.28 (48.07 to 100.48)	102.39 (83.67 to 121.11)	-28.11 (-60.52 to 4.30)	0.087
**VAS of LBP**	767.56 (495.81 to 1039.31)	1029.81 (833.46 to 1226.16)	-262.25 (-598.87 to 74.38)	0.122
**VAS of LEG**	886.49 (628.94 to 1144.05)	1168.39 (986.94 to 1349.84)	-281.90 (-603.16 to 39.36)	0.083
**ODI**	657.77 (489.71 to 825.84)	708.24 (590.86 to 825.62)	-50.46 (-258.05 to 157.12)	0.624
**EQ5D5L**	19.85 (18.26 to 21.43)	19.56 (18.44 to 20.68)	0.29 (-1.65 to 2.23)	0.763
**PCS**	1156.81 (1063.89 to 1249.74)	1096.77 (1032.53 to 1161.01)	60.04 (-54.34 to 174.41)	0.293
**MCS**	1275.87 (1172.61 to 1379.14)	1276.27 (1201.97 to 1350.58)	-0.40 (-128.06 to 127.25)	0.995

Table S10. MRI findings for the two groups at screening and Week 27.

		Screening			Week 27		
		**Non-PHM (n=10)**	**PHM (n=18)**	**P value**	**Non-PHM (n=10)**	**PHM (n=18)**	**P value**
Desiccated + Bulging	Yes	10 (100.00%)	14 (77.78%)	0.265	10 (100.00%)	13 (72.22%)	0.128
	No	0 (0.0%)	4 (22.22%)		0 (0.0%)	5 (27.78%)	
Protrusion	Yes	7 (70.00%)	16 (88.89%)	0.315	6 (60.00%)	17 (94.44%)	0.041
	No	3 (30.00%)	2 (11.11%)		4 (40.00%)	1 (5.56%)	
Extrusion	Yes	3 (30.00%)	5 (27.78%)	1	3 (30.00%)	3 (16.67%)	0.634
	No	7 (70.00%)	13 (72.22%)		7 (70.00%)	15 (83.33%)	
Sequestration + Migration	Yes	-	-		-	-	
	No	10 (100.00%)	18 (100.00%)		10 (100.00%)	18 (100.00%)	

*Fisher's exact test.

Table S11. Adverse events reported by the two groups.

Group	Lowest Level Term (LLT)	Severity	Serious adverse events	Causality
PHM	Headaches	Mild	No	Possibly related
Non-PHM	Stomatitis	Mild	No	Definitely not related
Non-PHM	Common cold	Mild	No	Definitely not related
PHM	Common cold	Mild	No	Definitely not related
PHM	Adhesive capsulitis	Moderate	No	Definitely not related
PHM	Headaches	Moderate	No	Definitely not related
PHM	COVID-19	Moderate	No	Definitely not related
PHM	Neck pain	Moderate	No	Definitely not related
PHM	Shoulder pain	Moderate	No	Definitely not related
